# Zebrafish ProVEGF-C Expression, Proteolytic Processing and Inhibitory Effect of Unprocessed ProVEGF-C during Fin Regeneration

**DOI:** 10.1371/journal.pone.0011438

**Published:** 2010-07-02

**Authors:** Abdel-Majid Khatib, Rachid Lahlil, Nathalie Scamuffa, Marie-Andrée Akimenko, Sylvain Ernest, Abdderahim Lomri, Claude Lalou, Nabil G. Seidah, Bruno O. Villoutreix, Fabien Calvo, Geraldine Siegfried

**Affiliations:** 1 INSERM, UMRS940, Equipe Avenir. Institut de Génétique Moléculaire, Hôpital St-Louis, Université Paris 7, Paris, France; 2 INSERM U 770, Kremlin-Bicetre, France; 3 Ottawa Health Research Institute, Ottawa, Ontario, Canada; 4 INSERM, U 784, Ecole Normale Supérieure, Paris, France; 5 INSERM U 606, Université Paris 7, Lariboisière Hospital, Paris, France; 6 Clinical Research Institute of Montreal, IRCM, Montreal, Quebec, Canada; 7 INSERM U648, Université Paris 5, Paris, France; University of Birmingham, United Kingdom

## Abstract

**Background:**

In zebrafish, vascular endothelial growth factor-C precursor (proVEGF-C) processing occurs within the dibasic motif HSIIRR_214_ suggesting the involvement of one or more basic amino acid-specific proprotein convertases (PCs) in this process. In the present study, we examined zebrafish proVEGF-C expression and processing and the effect of unprocessed proVEGF-C on caudal fin regeneration.

**Methodology/Principal Findings:**

Cell transfection assays revealed that the cleavage of proVEGF-C, mainly mediated by the proprotein convertases Furin and PC5 and to a less degree by PACE4 and PC7, is abolished by PCs inhibitors or by mutation of its cleavage site (HSIIRR_214_ into HSIISS_214_). *In vitro*, unprocessed proVEGF-C failed to activate its signaling proteins Akt and ERK and to induce cell proliferation. *In vivo*, following caudal fin amputation, the induction of VEGF-C, Furin and PC5 expression occurs as early as 2 days post-amputation (dpa) with a maximum levels at 4–7 dpa. Using immunofluorescence staining we localized high expression of VEGF-C and the convertases Furin and PC5 surrounding the apical growth zone of the regenerating fin. While expression of wild-type proVEGF-C in this area had no effect, unprocessed proVEGF-C inhibited fin regeneration.

**Conclusions/Significances:**

Taken together, these data indicate that zebrafish fin regeneration is associated with up-regulation of VEGF-C and the convertases Furin and PC5 and highlight the inhibitory effect of unprocessed proVEGF-C on fin regeneration.

## Introduction

Increased interest in using zebrafish as a model organism has led to a resurgence of fin regeneration studies. This has allowed for the identification of a large number of gene families, including signaling molecules and transcription factors expressed during tissues regeneration. The ability to regenerate multiple organs is limited to certain species, such as specific urodele amphibians and teleost fishes. While there is a widespread interest in regeneration [1], [2] especially given the recent emphasis on stem cell research, functional understanding of the regeneration process remains limited [1], [2]. When severely damaged or partially amputated, fins and heart of zebrafish are able of undergo self-restoration [1]–[3]. This complex process requires precise coordination between cell proliferation, differentiation, morphogenesis and pattern formation [1], [2]. Usually, cell proliferation and differentiation are controlled by a series of specific mitogenic and antimitogenic signals that drive multiple pathways within cells. Some of these signaling pathways implicate adhesion molecules, proteases, growth factors, as well as receptors. Lately, mammalian vascular endothelial growth factor-C (VEGF-C) was reported to be involved in the processes of lymphangiogenesis that results in the formation of a vascular network, which plays a pivotal role in the immune defense of vertebrates, as well as in the progression of cardiovascular diseases and tumor angiogenesis [4]. VEGF-C is secreted as a disulfide-bonded homodimer that is proteolytically processed from its precursor polypeptide proVEGF-C [5], [6]. VEGF-C signaling is carried out through activation of specific membrane protein-kinase receptors VEGF/R2 and VEGF/R3. Following proteolytic processing, VEGF-C binds to VEGF/R3 and mediates lymphangiogenesis, while its binding to VEGF/R2 promotes angiogenesis [5]–[7]. The existence of lymphatic system in zebrafish was also reported, requiring VEGF-C signaling for its maintenance and homeostasis [8]. Indeed, expression of VEGF-C was reported to be crucial for the development of lymphatic and vascular zebrafish systems [8]. Following examination of the amino acid sequence of zebrafish proVEGF-C precursor (NCBI sequence data base; AF466147), we detected a motif containing a pair of basic amino acids (aa), QHSIIRR_214_, reminiscent of the motifs recognized by the basic aa-specific proprotein convertases (PCs), suggesting the implication of these proteases in zebrafish proVEGF-C processing and activation. Previously, members of PC-family namely: Furin, PC1/3, PC2, PC4, PACE4, PC5/6 and PC7 have been reported to be involved in the processing of various substrates at basic residues within the general motif (K/R)-(X)_n_-(K/R)↓ where n = 0, 2, 4 or 6 and X is any amino acid except Cys, [9]-[12]. Usually most PCs cleave their substrates at pairs of basic amino acids, but several substrates; with monobasic sites are also cleaved [9]–[12]. Some PCs, such as PC1/3 and PC2, are sorted and activated in the regulated secretory pathway and thus process protein precursors whose secretion is regulated. In contrast, the trans-membrane proteins Furin, and PC5B cycle between the cell surface and the *trans* Golgi Network (TGN) and are involved in the processing of precursor proteins found in the constitutive secretory pathway [9]–[12]. α1-PDX was initially showed to be a potent inhibitor of various substrates cleavage by Furin in different species and subsequently demonstrated to also inhibit all PCs involved in processing within the constitutive secretory pathway [9]–[12]. To date, the prosegments are the only naturally occurring intracellular PCs inhibitors found in the constitutive secretory pathway. Although these prosegments were reported to be very potent inhibitors for their associated proteases, they were found not to be completely selective for their cognate enzymes and can inhibit other PCs [9]–[12]. The cleavage of some of the PCs substrates is crucial for the mediation of their functions such as in the case of IGF-1 receptor [12], [13]. On the other hand, the unprocessed forms of several molecules are biologically active, e.g., the integrin α4β1 [14], and in certain cases their processed forms are inactive such as endothelial lipase [15], FGF-23 [16] and MMP-2 [17], or mediate an opposite biological action through specific receptors, e.g., various neutrophins [18]. Previously, VEGF-C was reported to be processed in mammals by members of the PCs, however the importance of its processing in the mediation of its signaling pathways Akt and ERK activation and function in zebrafish is unknown. During fin regeneration, various steps are required: starting with healing based on the formation of a multistratified epidermal layer followed by the formation of a mass of pluripotent cells known as blastema. The latter are involved in the morphological growth and complete fin restoration [1], [2]. Since ontogenesis and fin regeneration (mediated by the blastema progenitor cells) were described to have similar molecular and cellular mechanisms [19], in the present study we used the ZF4 cells a fibroblast like cell line derived from 1-day-old zebrafish embryos to explore the role of proVEGF-C processing in Akt and ERK activation and cell proliferation *in vitro*. *In vivo*, we assessed the effect of unprocessed pro-VEGF-C during zebrafish fin regeneration.

## Materials and Methods

### Cell culture and transfections

The Furin-deficient LoVo [20] and zebrafish embryos-derived ZF4 cells [21] (ATCC) were maintained with F12 and DMEM/F12 media, respectively, supplemented with 10% FCS, 100 units/ml penicillin, and 100 µg/ml streptomycin (Invitrogen). The LoVo cells were transiently co-transfected with zebrafish VEGF-C construct cloned in the pSecTagB vector which contains an immunoglobulin κ signal sequence and C-terminal Myc and His6 tags [7] and empty pIRES2-EGFP vector or with pIRES2-EGFP vector that expresses full-length PCs: Furin, PACE4, PC5/6 (herein called PC5), or PC7 cDNAs [9]–[12]. The ZF4 cells were transiently co-transfected with pSecTagB vector containing zebrafish VEGF-C cDNA and empty pIRES2-EGFP or pIRES2-EGFP vector expressing the PCs inhibitors: the prosegment of Furin (ppFurin) [9]–[12] and the variant of α1-antitrypsin (α1-PDX) [22]. In other experiments ZF4 cells were transiently transfected with vector expressing either wild-type or mutated VEGF-C cDNAs (HSIIRR to HSIISS) generated by PCR mutagenesis. Transfections were carried out using the Effectene transfection reagent (Qiagen) according to manufacturer's instructions.

### Real-time PCR

Total RNA was subjected to cDNA synthesis using the Superscript first strand cDNA synthesis system (Invitrogen). The relative quantification of specific mRNAs was performed by real-time PCR using the StepOnePlus™ Real-Time PCR System and Power SYBR Green PCR Master Mix (Applied Biosystems) according to the manufacturer's instructions. In brief, the mixture of the reaction consists in 20 µl total volume of 2 µl of cDNA, 2 x QuantiTect SYBER Green PCR Master Mix, and 0.5 µM of the forward and reverse primers indicated in Table 1. PCR reaction was performed at 94°C for 15 s and at 60°C for 1 min during 40 cycles. The transcription of β-actin that was evaluated in each sample was used as endogenous control.

**Table 1:** Primers used for Real-time PCR analysis.

	Forward primer	Reverse primer
**Furin**	ACCTGGCTGTTCACCTCATC	TCAATCTCCAGGGTCCATTC
**PC5**	ATCCATCATGTGCCGAAAAT	GCACAGGTGGCACAAGTAGA
**VEGFR2**	GGTGAAGAAGGACGATGAGG	ACAGGAATGTTGCTGCTGCT
**VEGFR 3**	AAAAAGGGTTGGTCGATTCC	GGCGAGTCTTCAGGAAACAG
**VEGF-C**	GGCCTCAACAGAGCTTCAAC	TCTCTTGGGGTCCACGTTAC
**Actin**	CACAGATCATGTTCGAGACCT	AGGGCGTAACCCTCGTAGAT

### Western Blotting

Cells were lysed in phosphate-buffered saline (PBS) containing 2% Nonidet P-40 and protease inhibitors (Roche). Media or lysates were subjected to SDS-polyacrylamide gel electrophoresis on 8% gels and proteins were blotted onto nitrocellulose membranes. The primary antibodies used were anti-Myc (Cell Signaling), anti-V5 (Sigma). Primary antibodies were revealed by horseradish peroxidase-conjugated secondary antibodies (Amersham, Pharmacia Biotech) and Enhanced chemiluminescence (ECL+Plus, Amersham Pharmacia Biotech) according to the manufacturers' instructions.

### Measurement of proprotein convertases activity

PCs activity in cells was assessed by evaluating their ability to digest the universal PCs substrate, the fluorogenic peptide pERTKR-MCA, as previously described [23]. In brief, cell extracts were incubated with pERTKR-MCA (100 µmol/L) during the indicated time periods in the presence of 25 mmol/L Tris, 25 mmol/L methyl-ethane-sulfonic acid, and 2.5 mmol/L CaCl_2_, pH 7.4 at 37°C and the fluorometric measurements were performed using a spectrofluorometer (FLUOstar OPTIMA; BMG Labtech) [23]. The general PCs-inhibitor decanoyl-RVKR-cholromethyl ketone was obtained from Calbiochem and recombinant Furin form Sigma.

### Akt and ERK Tyrosine Phosphorylation

Confluent ZF4 cells grown in 75-cm2 flasks were maintained in serum-free DMEM for 24–48 h and incubated with media derived from LoVo cells transfected with empty vector or vector containing the indicated PCs for 2 min at 37°C. Cells were washed twice in ice-cold PBS and lysed with 500 µl/dish in lysis buffer (50 mM HEPES (pH 7.6), 150 mM NaCl, 1% Triton X-100, 2 mM vanadate, 100 mM NaF, and 0.40 mg/ml phenylmethylsulfonyl fluoride) [13]. Equal amounts of proteins (1 mg) were analyzed by Western blotting for Akt and ERK phosphorylation using an anti-phospho-Akt and anti-phospho-ERK, respectively. The anti-Akt and anti-ERK (Cell Signaling) were used for data normalization.

### Proliferation assay

ZF4 cells were plated in triplicate on 96 wells plate (5.10^3^/well) under serum free conditions for 24 h. The starved cells were then incubated with conditioned media derived from LoVo cells transfected with empty pSecTagB and pIRES2-EGFP vectors, or co-transfected with VEGF-C construct in pSecTagB vector and empty pIRES2-EGFP vector or co-transfected with VEGF-C construct in pSecTagB vector and Furin construct in pIRES2-EGFP. In other experiments, LoVo and ZF4 cells were transfected with wild type or mutant VEGF-C (HSIIRR to HSIISS) plasmid constructs. Proliferation level in cells was evaluated using the Cell Titer96 non-radioactive cell proliferation assay kit (Promega) according to manufacture's protocol.

### Animals and fin regeneration assay

Wild-type zebrafish (Danio rerio) and the transgenic zebrafish *Fli*-EGFP-Tg were purchased from the ZIRC fish center (Oregon) and were housed under standard conditions [24] in a core facility and water temperature was maintained at 28.5°C. Ethical approval for all animal studies was obtained from the Institutional Animal Care and Use Committee of the INSERM Institute and the University of Paris7 in accordance with the National Advisory Committee for Laboratory Animal Research Guidelines licensed by the French Authority. For zebrafish fin regeneration assay, adult fish of at least 10 weeks were anesthetized by addition of 0.6 mM tricaine (ethyl-m-aminobenzoate) to water. Caudal fins were amputated at a level proximal to the first bifurcation of the bony rays using razor blades. Animals were allowed to regenerate for various times at 28.5°C depending on the experiment. In other experiments, adult zebrafish were anesthetized in tricaine and were injected with vectors containing wild-type or mutant VEGF-C cDNA into the dermal skeleton of caudal fin using microcapillaries, as we previously described [24]. In brief, following injections, 10 consecutive 50-ms electric pulses, at 15 V with a 1-second pause between pulses, were applied via a pair of electrode disks (7 mm in diameter). Twenty-four hours post-injection, caudal fins were amputated at a level proximal to the first bifurcation with a scalpel, and fish were returned to a 28.5°C tank.

### Immunohistochemistry

For VEGF-C, Furin and PC5 detection, fin regenerates derived from the transgenic zebrafish *Fli*-EGFP-Tg were incubated overnight at 4°C with anti-VEGF-C, anti-Furin or anti-PC5, respectively. On the following day, fins were washed and were incubated with secondary antibody. Negative controls were directly incubated with the secondary antibody. Photographs were taken on a Zeiss Axioplan 2 Digital Imaging Microscope (Carl Zeiss Microscopy).

## Results

### Zebrafish proVEGF-C processing by the PCs

**Figure 1:**
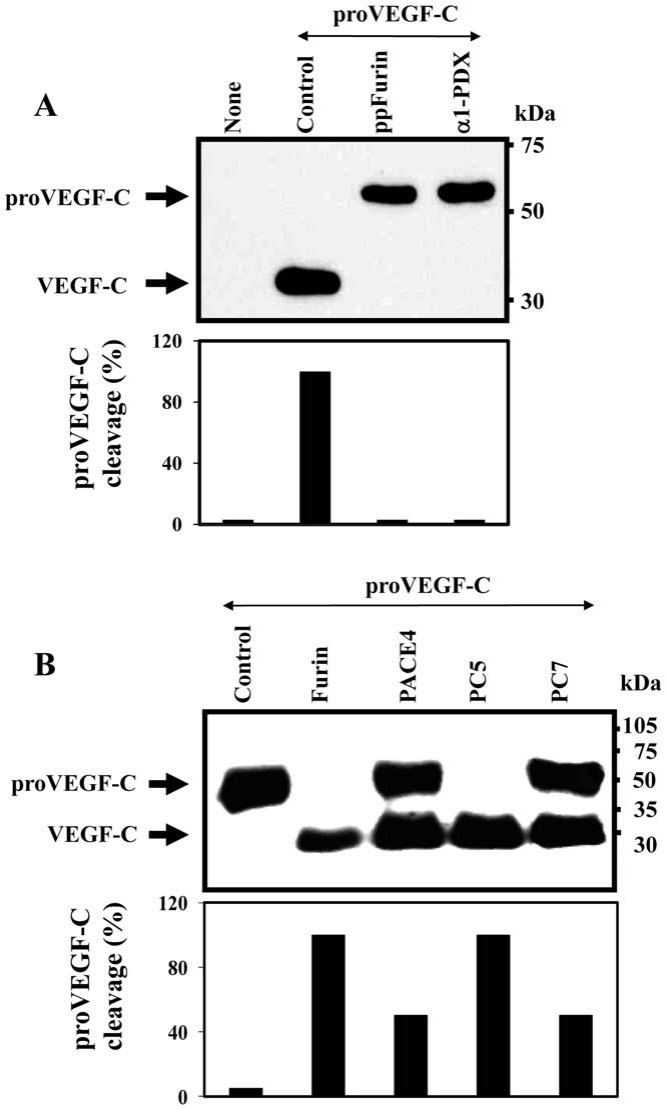
Processing of zebrafish proVEGF-C. (A) Processing of proVEGF-C was analyzed by Western blotting in zebrafish ZF4 cells transiently cotransfected with pSecTagB (Myc tag) vector containing the zebrafish proVEGF-C cDNA and the empty pIRES2-EGFP vector (Control) or pSecTagB expressing proVEGF-C and pIRES2-EGFP containing the PCs inhibitors; ppFurin or α1-PDX. Results of band intensities are shown in the bar graph that were deduced from the ratio of VEGF-C/(pro-VEGF-C + VEGF-C). Results are representative of three experiments. (B) The processing of proVEGF-C was analyzed by Western blotting using anti-Myc antibody on media derived from PCs-deficient LoVo cells transiently cotransfected with either pIRES2-EGFP vector alone and pSecTagB (Myc tag) containing the zebrafish VEGF-C cDNA (Control) or pIRES2-EGFP vector containing Furin, PACE4, PC5 or PC7 cDNA. The corresponding percentages of band intensities were deduced from the ratio of VEGF-C/(proVEGF-C+VEGF-C). Results are representative of three experiments.

In order to assess the efficiency of PC-inhibitors on zebrafish pro-VEGF-C processing, ZF4 cells were co-transfected with plasmid constructs expressing VEGF-C and each of the PC-inhibitors, including the PC-prosegment of Furin (ppFurin) [10] and the variant of α1-antitrypsin (α1-PDX) [22]. Processing of proVEGF-C was analyzed by immunoblotting. ZF4 cells transfected with the proVEGF-C construct revealed complete processing of proVEGF-C, as evidenced by the absence of unprocessed proVEGF-C (Fig. 1A), suggesting the presence of a PC-like activity in these cells. Indeed, co-transfection of ZF4 cells with proVEGF-C and the PC inhibitors α1-PDX or ppFurin, completely blocked this processing (Fig. 1A).

**Figure 2:**
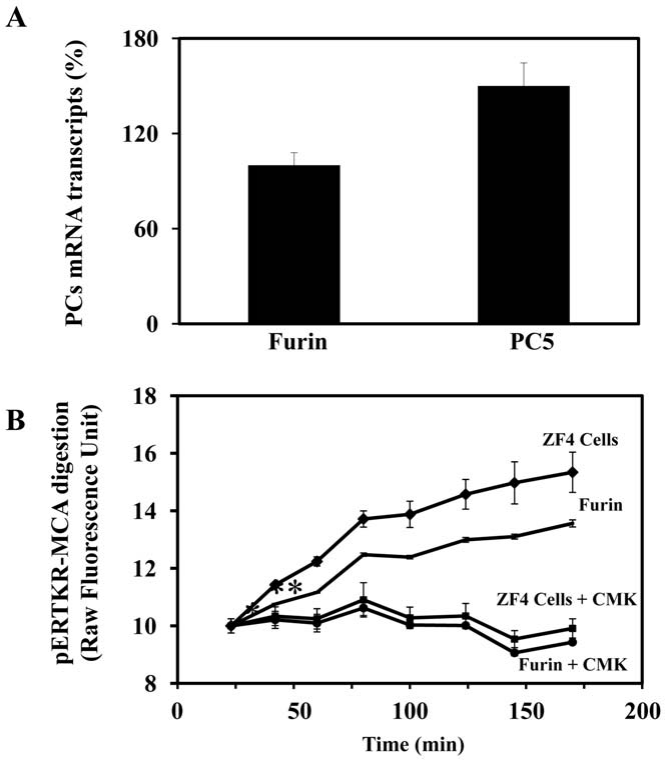
PCs expression and activity in ZF4 cells. (A) Following total RNA extraction from 10^4^ x ZF4 cells, real-time PCR analysis was performed using specific primers for Furin, PC5 or β-actin zebrafish as described in Material and Methods. During PCR, the transcription of β-actin that was evaluated in each sample was used as endogenous control. Results are shown in the bar graph and are expressed as the percentage of the indicated transcripts relative to Furin transcript (100%). Data are shown as means ± S.E of three experiments performed in duplicate. (B) PCs activity in ZF4 cells was assessed by evaluating the cells protein extract ability to digest the universal PCs substrate, the fluorogenic peptide pERTKR-MCA at the indicated time periods. Digestion of pERTKR-MCA by recombinant Furin (2 unit/µl) is given for comparison. As can be seen, the PCs inhibitor peptidyl chloromethyl ketones (CMK) (10 µM) reduced dramatically the PCs activity in ZF4 cells and the activity of recombinant Furin. Results are representative of two experiments performed in triplicate and data are mean ± S.E. *p<0.005; **p<0.0001.

To date, four members of the basic aa-specific PC-family namely, Furin, PACE4, PC5, and PC7 have been implicated in the processing of precursor molecules in cells lacking secretory granules, within a pathway known as the constitutive secretory pathway [9]–[12]. To identify which of these PCs are involved in zebrafish proVEGF-C processing; a zebrafish proVEGF-C cDNA construct and plasmids expressing each of these proteases were transiently co-transfected in the Furin-deficient LoVo cells [20]. These cells were previously reported to be unable to process many proprotein convertases precursors, due to their lack of Furin activity [20], [25]. After transfection, media of LoVo cells were analyzed for proVEGF-C processing by immunoblotting. Analysis of media derived from LoVo cells co-transfected with proVEGF-C and empty vector (Control) revealed only one immunoreactive protein with an apparent molecular mass of ∼59 kDa, corresponding to the intact zebrafish proVEGF-C precursor (Fig. 1B). Co-transfection of these cells with zebrafish proVEGF-C and Furin, PACE4, PC5 or PC7 plasmid constructs revealed that the expression of these convertases restored the ability of LoVo cells to process zebrafish proVEGF-C, as revealed by the reduction in the level of the immunoreactive precursor and the concomitant appearance of a product of ∼29 kDa, corresponding to the mature form of VEGF-C. Under these conditions, only Furin and PC5 completely cleaved zebrafish proVEGF-C into mature VEGF-C, while PACE4 and PC7 can only partially process this precursor (Fig. 1B). Using real-time PCR analysis Furin and PC5 were found to be expressed in ZF4 cells with a predominance of PC5 expression (Fig. 2A). Similarly, analysis of PC activity in these cells using an *in vitro* enzymatic digestion assay confirmed the presence of high PCs activity, that is inhibited by 10 µM of the peptidic PCs-inhibitor dec-RVKR-CMK (Fig. 2B) [10], [23].

### Requirement of proVEGF-C processing in VEGF-C-induced Akt and ERK tyrosine phosphorylation and cell proliferation

**Figure 3:**
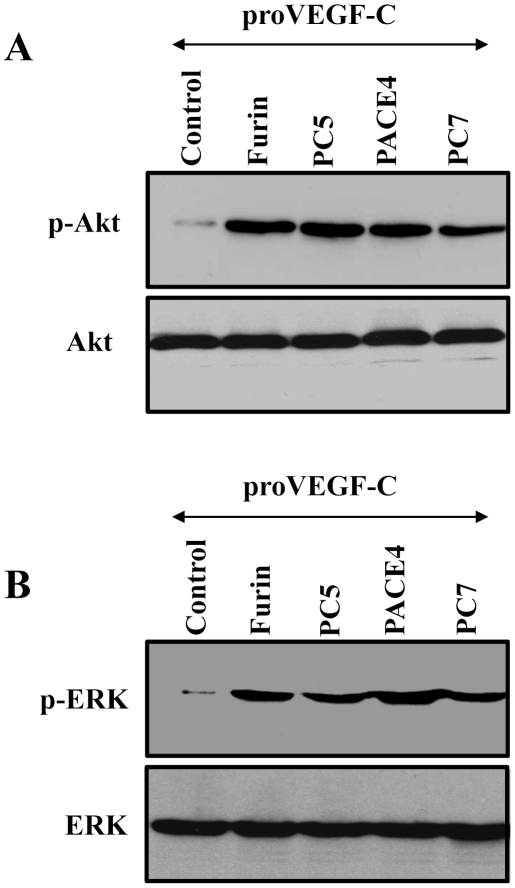
Requirement of the PCs in VEGF-C-mediated Akt and ERK phosphorylation in ZF4 cells. Confluent ZF4 cells were serum starved for 24–48 h and then treated for 2 min at 37°C with media derived from LoVo cells transiently cotransfected with empty vectors and pSecTagB vector containing proVEGF-C construct (Control) or pSecTagB vector containing proVEGF-C cDNA and pIRES2-EGFP vector expressing full-length Furin, PACE4, PC5 or PC7 cDNAs. Equal amounts of cell lysates were subjected to Western blotting using an anti-phospho-Akt (A), or an anti-phospho-ERK (B). The anti-Akt and anti-ERK were used for data normalization. Results are representative of three experiments.

**Figure 4:**
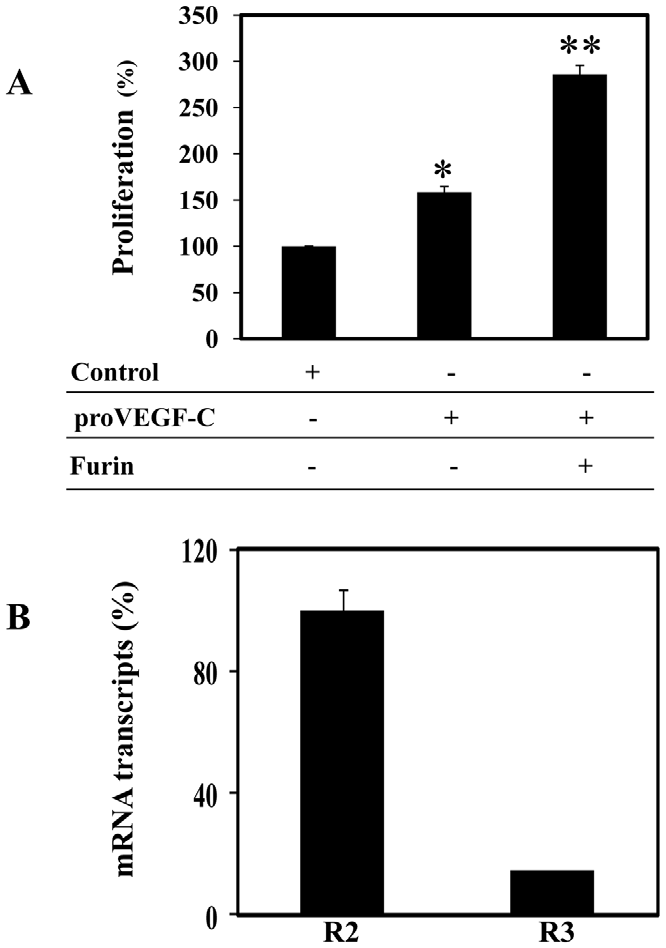
Effect of proVEGF-C processing on ZF4 cells proliferation. (A) ZF4 cells were serum deprived overnight and then treated for 24 h with media derived from LoVo cells transiently cotransfected with empty vectors (Control) or empty vector and vector containing proVEGF-C construct or vector expressing proVEGF-C cDNA and vector expressing Furin cDNA. Cell proliferation was assessed using Cell Titer96 non-radioactive cell proliferation assay. Results are shown as means ± S.E. of three experiments performed in triplicate. (B) Total RNA derived from ZF4 cells was subjected to real-time PCR analysis using specific primers for the zebrafish VEGF-C receptors R2, R3 or β-actin. During PCR, the transcription of β-actin that was evaluated in each sample was used as endogenous control. Results are shown in the bar graph and are expressed as the ratio of the indicated transcripts relative to R2 transcript assigned to 100%. Results are shown as means ± S.E. of three experiments performed in duplicate.

Like the other VEGFs, VEGF-C elicits its biological activity through interactions with its transmembrane high-affinity receptors R2 or R3. Binding of VEGF-C ligand to these receptors results in their autophosphorylation [26]. In turn, the phosphorylated receptors activate an enzymatic cascade that induces the activation of various phosphorylating enzymes including Akt and ERK, and ultimately triggers cell division and other cellular functions [27]. To examine whether proVEGF-C processing by PCs is required for the mediation of Akt, ERK activation, conditioned media derived from the Furin-deficient LoVo cells transfected with proVEGF-C alone or co-transfected with Furin, PC5, PACE4 or PC7 plasmids were tested for Akt and ERK tyrosine phosphorylation in ZF4 cells. Media derived from LoVo cells transfected with proVEGF-C alone, failed to induce significant Akt (Fig. 3A, Control) and ERK (Fig. 3B, Control) tyrosine phosphorylation. In contrast, incubation of ZF4 cells with media derived from LoVo cells co-transfected with VEGF-C and Furin, PACE4, PC5 or PC7 induced both Akt and ERK tyrosine phosphorylation in these cells, emphasizing the importance of PCs in proVEGF-C processing and activation (Fig. 3). As can be seen, although PACE4 and PC7 were less efficient in the mediation of proVEGF-C processing (Fig.1B), the amount of the processed forms of VEGF-C generated in LoVo cells cotransfected with these PCs and proVEGF-C cDNAs was enough to induce Akt and ERK phosphorylation (Fig.3). Similarly, incubation of ZF4 cells with media derived from LoVo cells co-transfected with proVEGF-C and Furin constructs significantly induced cell proliferation as compared to media derived from LoVo cells transfected with empty vector (Control) or proVEGF-C alone (Fig. 4A). The observed induction of cell proliferation in the presence of media derived from LoVo cells transfected with vector that contains solely wild-type proVEGF-C is likely due to proVEGF-C maturation by PCs-like activity found on the surface of ZF4 cells. Indeed, previous studies reported that both Furin and PC5B possess in their amino acid sequence a *trans*-membrane domain that allows them to circle between the TGN and the cell surface and process their substrates therein [9]. Alternatively, this surface-processing of proVEGF-C may also be due to the presence of active PC5A at the surface of ZF4 cells through interaction with heparan sulfate proteoglycans [28]. Analysis of ZF4 cells by real-time PCR revealed that ZF4 cells express both VEGF-C receptors, namely VEGF/R2 and VEGF/R3 with a predominance expression of VEGF/R2 receptors (Fig. 4B).

### Induction of VEGF-C, Furin and PC5 during fin regeneration

**Figure 5:**
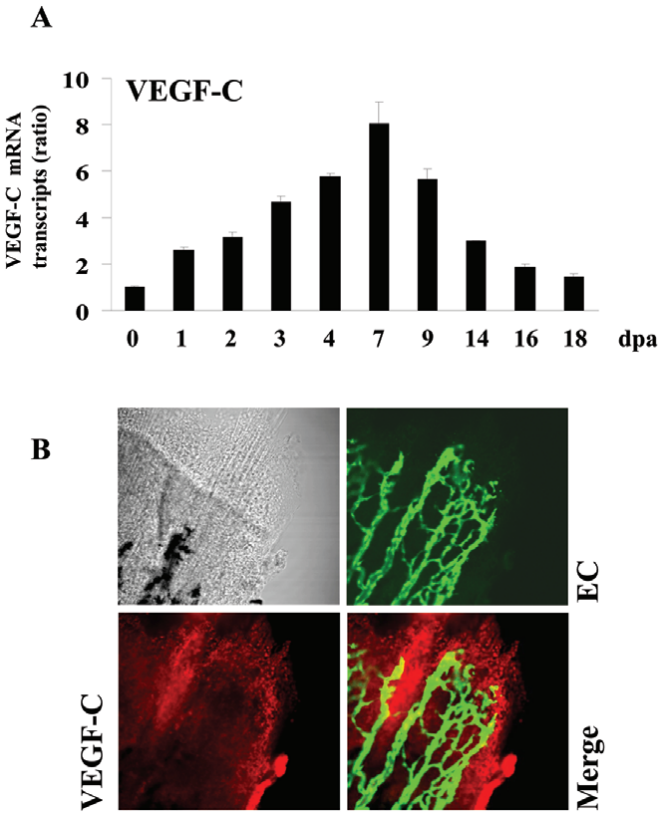
Expression of VEGF-C during fin regeneration. Adult zebrafish were anesthetized in tricaine and fins were cut using razor blades. (A) Total RNA was isolated from fins (15–20 fins per time point) with uncut fin as control (0 dpa) and analyzed by real-time PCR using specific primers for zebrafish VEGF-C or β-actin. Results are shown in the bar graph and are expressed as the ratio of the indicated transcripts relative to control (0 dpa). Results are shown as means ± S.E. of three experiments performed in triplicate. (B) Expression patterns of VEGF-C at 3 dpa were analyzed by immunofluorescence with an anti-VEGF-C (red signal) using fli-EGFP transgenic zebra fish that allows the visualisation of endothelial cells (EC) (green signal). The expression of VEGF-C was mainly localized to the apical growth zone of the regenerating fin (red signal, 25× objective).

**Figure 6:**
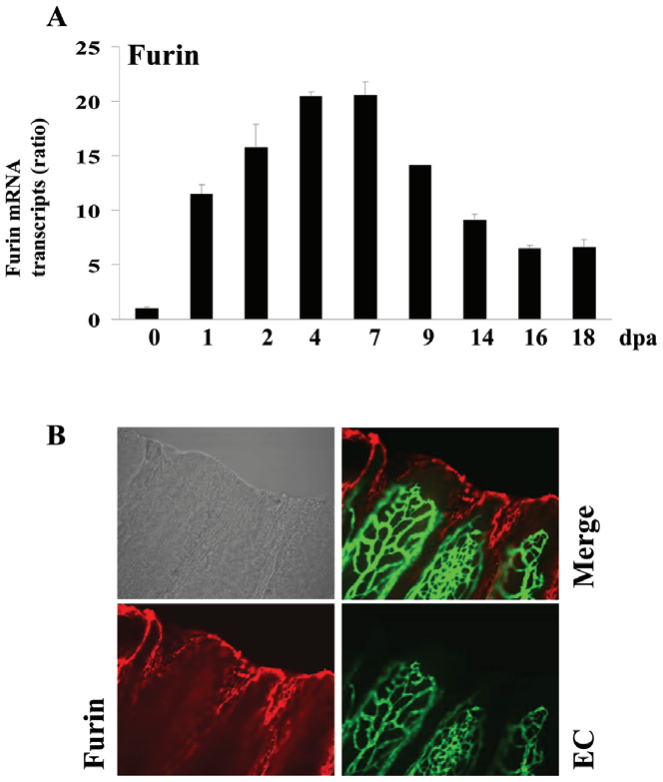
Expression of Furin during fin regeneration. (A) Total RNA was isolated from fins (15–20 fins per time point) and analyzed by real-time PCR using specific primers for zebrafish Furin or β-actin. Results are shown in the bar graph and are expressed as the ratio of the indicated transcripts relative to control (0 dpa). Results are shown as means ± S.E. of three experiments performed in triplicate. (B) Immunofluorescence analysis revealed that Furin is mainly localized to the apical growth zone of the regenerating fin (red signal, 25× objective).

**Figure 7:**
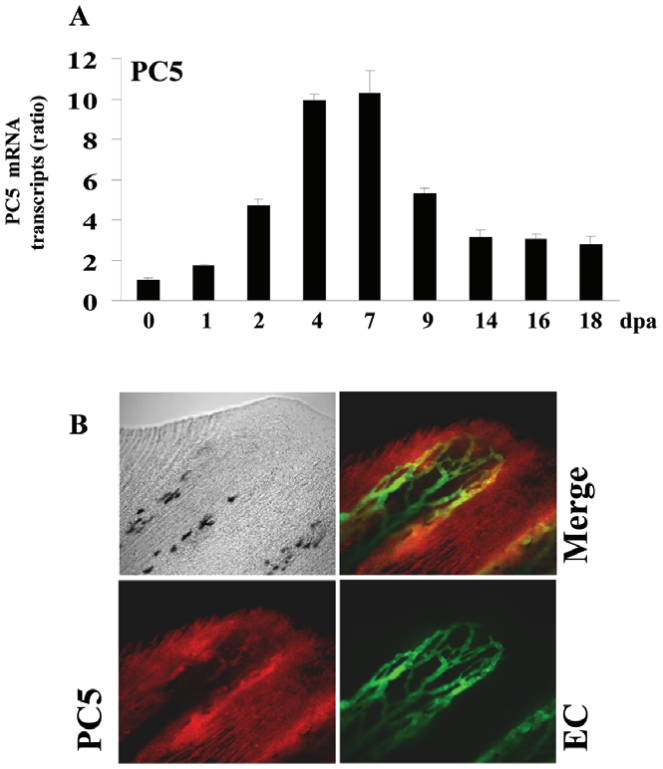
Expression of PC5 during fin regeneration. (A) Total RNA was isolated from fins (15-20 fins per time point) and analyzed by real-time PCR using specific primers for zebrafish PC5 or β-actin. Results are shown in the bar graph and are expressed as the ratio of the indicated transcripts relative to control (0 dpa). Results are shown as means ± S.E. of three experiments performed in triplicate. (B) Immunofluorescence analysis revealed that PC5 is expressed in all area of the regenerating fin and low signal was observed on the vessels (red signal, 25× objective).

To determine whether VEGF-C and its major convertases Furin and/or PC5 expression are induced during fin regeneration, zebrafish caudal fins were amputated and the expression of these molecules was analyzed using real-time PCR during 18 dpa (18 dpa corresponds to the almost complete regenerated fin). Only low constitutive levels of VEGF-C (Fig. 5A), Furin (Fig. 6A) and PC5 (Fig. 7A) were detected in fins obtained from controls, not-amputated zebrafish fins. Following amputation, mRNA transcripts of these molecules began to increase within 1–2 dpa, reaching maximal levels at 4–7 dpa and declined thereafter (Figs. 5A, 6A, 7A). To explore the expression of VEGF-C, Furin and PC5 at the protein levels during fin regeneration we used the transgenic zebrafish *(fli1:EGFP)^y1^* that expresses EGFP in all endothelial cells and allow easy visualization of the vasculature in fins [29]. Using specific VEGF-C antibody, immunohistochemistry analysis of regenerated fins at 31 dpa confirmed the expression of VEGF-C at the protein level (Fig. 5B). Interestingly, high level of VEGF-C was detected in the periphery of the regenerating area that is constituted by the pluripotent blastema cells and only weak VEGF-C expression was detected in the vessels. Similarly, using specific Furin and PC5 antibodies, immunohistochemistry analysis of regenerated fins at 3 dpa revealed that although these convertases seemed to be expressed in all the regenerating area, high levels of these PCs, particularly, Furin was observed at the periphery of the regenerating area (Figs. 6B, 7B). Taken together, these data suggest that VEGF-C, Furin and PC5 expression induced at the apical growth zone; constituted with the progenitor blastema cells, may be implicated in the progressive replacement of the amputated structures of the fin.

### Mutation of proVEGF-C at its PCs cleavage site inhibited VEGF-C-induced *in vitro* cell proliferation

**Figure 8:**
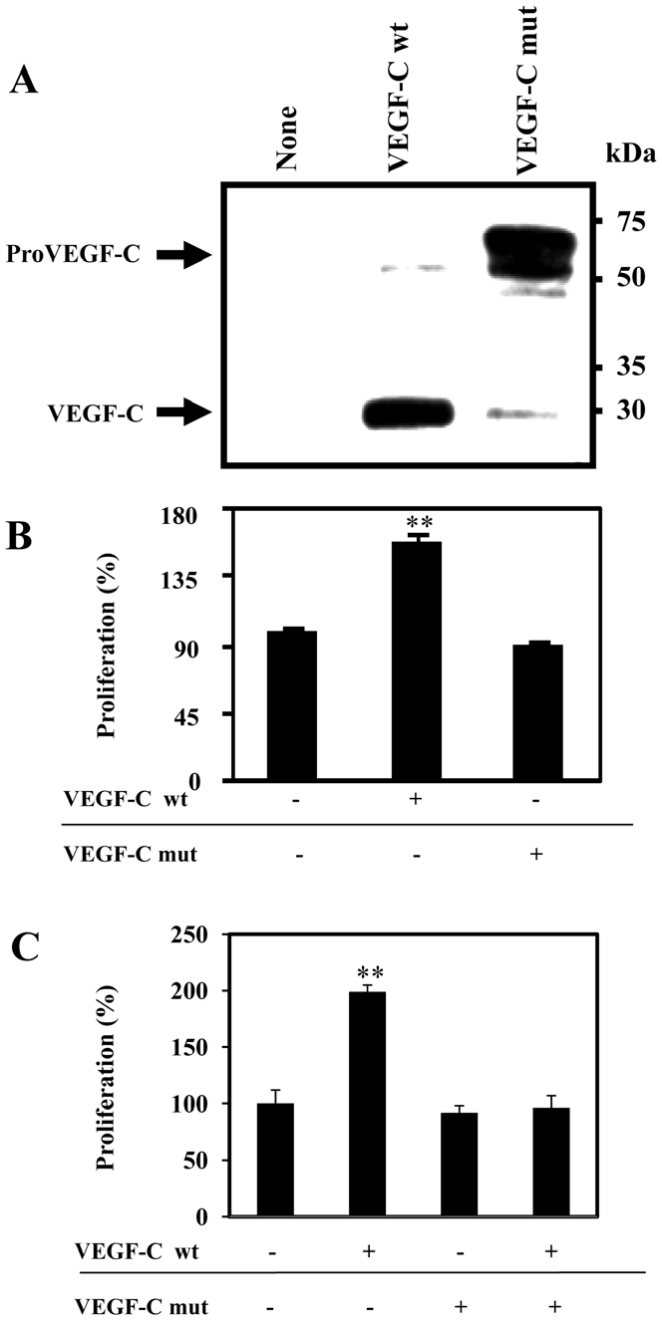
Uprocessed ProVEGF-C inhibits VEGF-C-induced ZF4 cells proliferation. (A) ZF4 cells were transiently transfected with empty vector (None), vector containing the wild-type (wt, HSIIRR) or mutated (mut, HSIISS) proVEGF-C cDNA. proVEGF-C processing was analyzed by western blot. Results are representative of three experiments. (B) ZF4 cells were serum deprived overnight and then treated for 24 h with media derived from LoVo cells transiently transfected with empty vectors (Control) or pSecTagB containing wild-type (wt) or mutated (mut) proVEGF-C cDNA. (C) ZF4 cells were serum deprived overnight and then treated or not for 6 h with media derived from LoVo cells transiently transfected with vector containing mut VEGF-C cDNA and incubated for 24 h with media derived from LoVo cells transfected with vector containing wt proVEGF-C cDNA. Cell proliferation was evaluated using the Cell Titer96 non-radioactive cell proliferation assay. Data are shown as means ± S.E. of three experiments performed in triplicate. *p<0.005; **p<0.0001.

We first generated a proVEGF-C mutant (proVEGF-C mut) in which the wild type PC cleavage site was modified by site directed mutagenesis from HSIIRR into HSIISS as previously described (6). Transfection of ZF4 cells with wild-type proVEGF-C resulted in its complete maturation, while expression of proVEGF-C mut was accumulated in the media as an unprocessed proVEGF-C form (Fig. 8A). Furthermore, incubation of ZF4 cells for 24h with media derived from LoVo cells transfected with proVEGF-C mut failed to enhance cell proliferation, as compared to those derived from LoVo cells transfected with a vector expressing wild-type proVEGF-C (Fig. 8B). Similarly, pretreatment of ZF4 cells with media derived from LoVo cells transfected with vectors expressing mutated unprocessed proVEGF-C for 6 h, prior their incubation with media derived from LoVo cells transfected with vector expressing wild-type proVEGF-C inhibited cell proliferation induced by media containing processed VEGF-C (Fig.8C).

### Unprocessed proVEGF-C inhibited zebrafish fin regeneration

**Figure 9:**
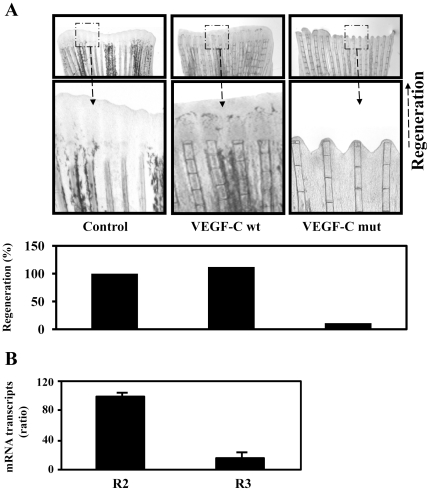
Uprocessed ProVEGF-C inhibits fin regeneration. (A) 24 h prior amputation of caudal fins (6 per group), empty vector (Control) or vector containing wild-type (wt) or mutant (mut) proVEGF-C constructs were injected in the fins and animals were allowed to regenerate at 28.5°C after fins amputation for 5 days. The microinjection of vector containing wild-type proVEGF-C had no effect on normal regeneration. In contrast, injection of the mutant proVEGF-C resulted in severe inhibition of fin regeneration. Results are representative of tree experiments. The corresponding percentages of regenerated area were deduced from the ratio of 100 x (fin surface regenerated in VEGF-Cwt)/(fin surface regenerated in Control) and 100 x (fin surface regenerated in VEGF-Cmut/fin surface regenerated in Control). (B) Total RNA derived from uncut fins was subjected to real-time PCR analysis using specific primers for the VEGF-C receptors R2, R3 or β-actin. During PCR, the transcription of β-actin that was evaluated in each sample was used as endogenous control. Results are shown in the bar graph and are expressed as the ratio of the indicated transcripts relative to R2 transcript assigned to 100%. Results are shown as means ± S.E. of three experiments performed in duplicate.

To evaluate the effect of unprocessed proVEGF-C on fin regeneration, wild-type or mutant proVEGF-C constructs were expressed following injection and electroporation in the caudal fin 24 h prior amputation (just proximal to the future level of amputation). When analyzed at 5 dpa, expression of the empty vector or the wild-type proVEGF-C had no effect on the normal regeneration process (Fig. 9A). In contrast, injection of vector expressing unprocessed proVEGF-C mut resulted in severe inhibition of fin regeneration (Fig. 9A) despite the presence of VEGF/R2 and VEGF/R3 in fins as revealed by real-time PCR analysis (Fig. 9B).

## Discussion

**Figure 10:**
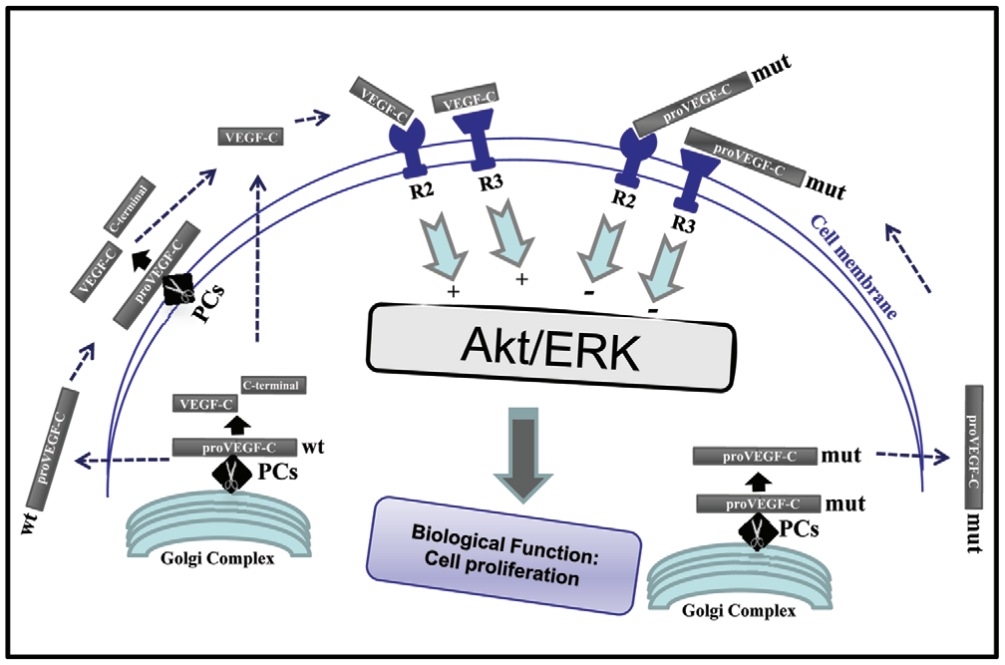
Schematic representation of the primary structure of proVEGF-C, its activating proteases and receptors R2 and R3. Upon synthesis and translocation into the Golgi network, proVEGF-C is processed at HSIIRR_214_, dividing it into N-terminal (VEGF-C) and C-terminal polypeptides. The cleavage of proVEGF-C can also occurs *via* cell surface-attached convertases (PCs). Interaction of mature VEGF-C (wt) with the receptors VEGF/R3 and VEGF/R2 leads to Akt/ERK activation and functions. Mutation of the cleavage site of proVEGF-C (mut) prevents Akt/ERK activation and cell proliferation.

VEGF-C was originally described as a specific growth factor for lymphatic vessels [30], [31], but later was also found to induce angiogenesis of blood vessels [30], [31] and its overexpression was linked to various cancers and metastasizing tumors [31]. Recently, the expression of VEGF-C in zebrafish was reported to be required during embryonic development where it mediates vasculogenesis and angiogenesis of the embryos [7]. In the present study, as summarized in Fig. 10, we demonstrated that the expression of zebrafish VEGF-C is upregulated during fin regeneration and the precursor proVEGF-C is proteolytically activated by several members of the PC-family that are also upregulated during fin regeneration. Additionally, we found that overexpression of unprocessed proVEGF-C in the amputated fins inhibited regeneration.

Upon synthesis of pre-proVEGF-C, the signal peptide is rapidly removed and proVEGF-C is then translocated to the Golgi network where other post-translational modifications occur [5] (Fig.10). Using the Furin-deficient cell line LoVo, we found that Furin and PC5 are the proVEGF-C convertases, while PACE4 and PC7 can process proVEGF-C to a lesser degree (Fig. 1). Similarly expressing of the PC general inhibitor α1-PDX or the prosegment of Furin (ppFurin) in the zebrafish cells ZF4 inhibited Pro-VEGF-C processing. Recently the processing of proVEGF-C was also found to be inhibited by other PCs inhibitors such as the case of a synthetic Furin-derived peptide that contains a highly reactive beta-turn-inducing and radical generating “enediynyl amino acid” (Eda) moiety [32]. Processing of proVEGF-C occurs at HSIIRR_214_, dividing it into an N-terminal (∼31 kDa) and cysteine-rich C-terminal (∼29 kDa) polypeptides [5], [6] (Fig.10). Previously, additional processing of mammalian VEGF-C was reported to remove the N-terminal propeptide and to generate the ∼21-kDa VEGF-C *via* an unknown secretory protease [5]. It is believed that the conversion of the ∼31-kDa VEGF-C (that binds only to VEGF/R3 receptor) to the ∼21-kDa form results in greatly enhanced affinity for both VEGF receptors R3 and R2 [5], [6] leading to Akt and ERK activation (Fig. 10) [27]. We found that unprocessed proVEGF-C failed to mediate Akt and ERK tyrosine phosphorylation as well as cell proliferation in ZF4 cells (Figs. 3, 4, 8). Similarly, to investigate the biological role of proVEGF-C processing in fin regeneration, we compared the effects of wild type and mutant proVEGF-C on fin regeneration. Our results demonstrate that over-expression of wild type proVEGF-C prior to fin amputation had no effect on regeneration. In contrast, expression of the mutant proVEGF-C under the same conditions completely blocked regeneration (Fig. 9A). Previous reports in other systems linked the inability of VEGF-C to stimulate cell proliferation to its weak mitogenic action compared with the other VEGFs [6], [26]. On the other hand, VEGF-C was reported to synergize with various factors, including VEGF and basic FGF signaling [33], [34] reported to be crucial during the processes of angiogenesis and regeneration in zebrafish [35]–[37]. In addition to the cooperative action of VEGF and VEGF-C, their common activation of VEGFR/2 was reported to constitute an important way to mediate their functions. Thus, VEGF-C and VEGF can both bind VEGF/R2, and probably displace each other. In addition, previous studies reported that unprocessed proVEGF-C was able to effectively displace mature VEGF-C from VEGF/R2 and VEGF/R3[5]. This may explain our findings of the inability of unprocessed proVEGF-C to induce Akt and ERK tyrosine phosphorylation in the embryonic zebrafish ZF4 cells that express the VEGF/R2 and VEGF/R3 receptors and the inhibitory effect of unprocessed pro-VEGF-C on the proliferation of processed VEGF-C-stimulated ZF4 cells (Figs 3, 4, 8). Thus proVEGF-C seems to behave as an antagonist to both receptor subtypes and to prevent their activation. In zebrafish, it is possible that the secretion of unprocessed proVEGF-C in the microenvironment of the regenerating fin may compete with active VEGF-C and VEGF produced by endothelial and hematopoietic cells within the regenerate, and hence antagonize the action of these factors on the formation of lymphatic and vascular vessels, required for tissue remodeling and regeneration.

Usually following fin injury, the regeneration process begins with wound healing and the formation of the blastema a mass of progenitor cells that form an apical growth zone with increased migration and proliferation activity required for the replacement of the amputated fins structures. These processes are associated with increased expression of several genes encoding secreted molecules that induce cell proliferation and migration around the wound [1], [2]. Secreted molecules previously reported to be originating from the wound, include growth factors such as FGF and PDGF-BB, metalloproteases such as MMPs and extracellular matrix proteins such as fibronectin [36]. In the present study, we found that VEGF-C and its activating convertases Furin and PC5 expression is also induced following fin injury (Figs. 5, 6, 7). VEGF-C expression was also previously reported to be induced in regenerating hearts of zebrafish [36]. The induction of VEGF-C in regenerating fin and heart, suggests the crucial role of this molecule in the processes of regeneration shared by the two systems. During fin regeneration, VEGF-C, Furin and PC5 are expressed as early as 1–2 dpa (Fig. 5, 6, 7). These data indicate that expression of VEGF-C and its PCs temporally overlap during regenerative outgrowth, reinforcing the functional link between these molecules. A temporal correlation between the expression of VEGF-C and Furin was previously observed in mice embryos [38], [39] suggesting a role for convertases in regulating VEGF-C functions during embryonic development. In conclusion, this study demonstrated that not only the expression of VEGF-C, Furin and PC5 is induced during fin regeneration, but that the proteolytic activation of proVEGF-C by Furin and PC5 is a critical step in VEGF-C-mediated signaling *in vitro* and unprocessed proVEGF-C inhibited fin regeneration. However, further investigations are required to elucidate the role of proVEGF-C processing in fin regeneration.

## References

[1] Akimenko MA, Mari-Beffa M, Becerra J, Geraudie J (2003). Old questions, new tools, and some answers to the mystery of fin regeneration.. Dev Dyn.

[2] Johnson SL, Bennett P (1999). Growth control in the ontogenetic and regenerating zebrafish fin.. Methods Cell Biol.

[3] Raya A, Consiglio A, Kawakami Y, Rodriguez-Esteban C, Izpisua-Belmonte JC (2004). The zebrafish as a model of heart regeneration.. Cloning Stem Cells.

[4] Alitalo K, Tammela T, Petrova TV (2005). Lymphangiogenesis in development and human disease.. Nature.

[5] Joukov V, Sorsa T, Kumar V, Jeltsch M, Claesson-Welsh L (1997). Proteolytic processing regulates receptor specificity and activity of VEGF-C.. Embo J.

[6] Siegfried G, Basak A, Cromlish JA, Benjannet S, Marcinkiewicz J (2003). The secretory proprotein convertases furin, PC5, and PC7 activate VEGF-C to induce tumorigenesis.. J Clin Invest.

[7] Ober EA, Olofsson B, Makinen T, Jin SW, Shoji W (2004). Vegfc is required for vascular development and endoderm morphogenesis in zebrafish.. EMBO Rep.

[8] Kuchler AM, Gjini E, Peterson-Maduro J, Cancilla B, Wolburg H (2006). Development of the zebrafish lymphatic system requires VEGFC signaling.. Curr Biol.

[9] Creemers JW, Khatib AM (2008). Knock-out mouse models of proprotein convertases: unique functions or redundancy?. Front Biosci.

[10] Bontemps Y, Scamuffa N, Calvo F, Khatib AM (2007). Potential opportunity in the development of new therapeutic agents based on endogenous and exogenous inhibitors of the proprotein convertases.. Med Res Rev.

[11] Lapierre M, Siegfried G, Scamuffa N, Bontemps Y, Calvo F (2007). Opposing function of the proprotein convertases furin and PACE4 on breast cancer cells' malignant phenotypes: role of tissue inhibitors of metalloproteinase-1.. Cancer Res.

[12] Khatib AM, Siegfried G, Prat A, Luis J, Chretien M (2001). Inhibition of proprotein convertases is associated with loss of growth and tumorigenicity of HT-29 human colon carcinoma cells: importance of insulin-like growth factor-1 (IGF-1) receptor processing in IGF-1-mediated functions.. J Biol Chem.

[13] Scamuffa N, Siegfried G, Bontemps Y, Ma L, Basak A (2008). Selective inhibition of proprotein convertases represses the metastatic potential of human colorectal tumor cells.. J Clin Invest.

[14] Bergeron E, Basak A, Decroly E, Seidah NG (2003). Processing of alpha4 integrin by the proprotein convertases: histidine at position P6 regulates cleavage.. Biochem J.

[15] Jin W, Fuki IV, Seidah NG, Benjannet S, Glick JM (2005). Proprotein convertases [corrected] are responsible for proteolysis and inactivation of endothelial lipase.. J Biol Chem.

[16] Fukumoto S (2005). Post-translational modification of Fibroblast Growth Factor 23.. Ther Apher Dial.

[17] Cao J, Rehemtulla A, Pavlaki M, Kozarekar P, Chiarelli C (2005). Furin directly cleaves proMMP-2 in the trans-Golgi network resulting in a nonfunctioning proteinase.. J Biol Chem.

[18] Lee R, Kermani P, Teng KK, Hempstead BL (2001). Regulation of cell survival by secreted proneurotrophins.. Science.

[19] Iovine MK (2007). Conserved mechanisms regulate outgrowth in zebrafish fins.. Nat Chem.

[20] Takahashi S, Kasai K, Hatsuzawa K, Kitamura N, Misumi Y (1993). A mutation of furin causes the lack of precursor-processing activity in human colon carcinoma LoVo cells.. Biochem Biophys Res Commun.

[21] Driever W, Rangini Z (1993). Characterization of a cell line derived from zebrafish (Brachydanio rerio) embryos.. In Vitro Cell Dev Biol Anim.

[22] Anderson ED, Thomas L, Hayflick JS, Thomas G (1993). Inhibition of HIV-1 gp160-dependent membrane fusion by a furin-directed alpha 1-antitrypsin variant.. J Biol Chem.

[23] Lalou C, Scamuffa N, Mourah S, Plassa F (2010). http://www.plosone.org/article/info%3Adoi%2F10.1371%2Fjournal.pone.0009992.

[24] Eyries M, Siegfried G, Ciumas M, Montagne K, Agrapart M (2008). Hypoxia-induced apelin expression regulates endothelial cell proliferation and regenerative angiogenesis.. Circ Res.

[25] Scamuffa N, Basak A, Lalou C, Wargnier A, Marcinkiewicz J (2008). Regulation of prohepcidin processing and activity by the subtilisin-like proprotein convertases Furin, PC5, PACE4 and PC7.. Gut.

[26] Joukov V, Pajusola K, Kaipainen A, Chilov D, Lahtinen I (1996). A novel vascular endothelial growth factor, VEGF-C, is a ligand for the Flt4 (VEGFR-3) and KDR (VEGFR-2) receptor tyrosine kinases.. Embo J.

[27] Petrova TV, Makinen T, Alitalo K (1999). Signaling via vascular endothelial growth factor receptors.. Exp Cell Res.

[28] Mayer G, Hamelin J, Asselin MC, Pasquato A, Marcinkiewicz E (2008). The regulated cell surface zymogen activation of the proprotein convertase PC5A directs the processing of its secretory substrates.. J Biol Chem.

[29] Lawson ND, Weinstein BM (2002). In vivo imaging of embryonic vascular development using transgenic zebrafish.. Dev Biol.

[30] Huang HY, Ho CC, Huang PH, Hsu SM (2001). Co-expression of VEGF-C and its receptors, VEGFR-2 and VEGFR-3, in endothelial cells of lymphangioma. Implication in autocrine or paracrine regulation of lymphangioma.. Lab Invest.

[31] Karkkainen MJ, Makinen T, Alitalo K (2002). Lymphatic endothelium: a new frontier of metastasis research.. Nat Cell Biol.

[32] Basak A, Khatib AM, Mohottalage D, Basak S, Kolajova M et al (2009). http://www.plosone.org/article/info%3Adoi%2F10.1371%2Fjournal.pone.0007700.

[33] Eggert A, Ikegaki N, Kwiatkowski J, Zhao H, Brodeur GM (2000). High-level expression of angiogenic factors is associated with advanced tumor stage in human neuroblastomas.. Clin Cancer Res.

[34] Pepper MS, Mandriota SJ, Jeltsch M, Kumar V, Alitalo K (1998). Vascular endothelial growth factor (VEGF)-C synergizes with basic fibroblast growth factor and VEGF in the induction of angiogenesis in vitro and alters endothelial cell extracellular proteolytic activity.. J Cell Physiol.

[35] Covassin LD, Villefranc JA, Kacergis MC, Weinstein BM, Lawson ND (2006). Distinct genetic interactions between multiple Vegf receptors are required for development of different blood vessel types in zebrafish.. Proc Natl Acad Sci U S A.

[36] Lien CL, Schebesta M, Makino S, Weber GJ, Keating MT (2006). Gene expression analysis of zebrafish heart regeneration.. PLoS Biol.

[37] Poss KD, Shen J, Nechiporuk A, McMahon G, Thisse B (2000). Roles for Fgf signaling during zebrafish fin regeneration.. Dev Biol.

[38] Lymboussaki A, Olofsson B, Eriksson U, Alitalo K (1999). Vascular endothelial growth factor (VEGF) and VEGF-C show overlapping binding sites in embryonic endothelia and distinct sites in differentiated adult endothelia.. Circ Res.

[39] Zheng M, Streck RD, Scott RE, Seidah NG, Pintar JE (1994). The developmental expression in rat of proteases furin, PC1, PC2, and carboxypeptidase E: implications for early maturation of proteolytic processing capacity.. J Neurosci.

